# A Good Cleansing Fluid

**Published:** 1878-06

**Authors:** 


A Good Cleansing Fluid.—The following is recommended for washing alpaca, camel's hair, and other woolen goods, and for removing marks made on furniture, carpets, rugs, etc.: Four ounces ammonia, four ounces white Castile soap, two ounces alcohol, two ounces glycerine, two ounces ether. Cut the soap fine, dissolve in one quart water over the fire, add four quarts water. When nearly cold, add the other ingredients. This will make nearly eight quarts, and will cost about seventy-five cents. It must be put in a bottle and stoppered tight. It will keep good any length of time. To wash dress goods, take a pail of lukewarm water and put in a teacupful of the fluid, shake around well in this, and then rinse in plenty of clean water, and iron on wrong side while damp. For washing grease from coat-collars, etc., take a little of the fluid in a cup of water, apply with a clean rag, and wipe with a second rag. It will make every woolen fabric look bright and fresh.—*Boston Journal of Chemistry.*

